# Assessing the Effect of Enzymatic Debridement on the Scar Quality in Partial-Thickness Burns to Deep Dermal Burns of the Hand: A Long-Term Evaluation

**DOI:** 10.3390/medicina60030481

**Published:** 2024-03-14

**Authors:** Wolfram Heitzmann, Alexandra Schulz, Paul Christian Fuchs, Jennifer Lynn Schiefer

**Affiliations:** Department of Plastic Surgery, Hand Surgery, Burn Center, University of Witten/Herdecke, Cologne-Merheim Medical Center (CMMC), 51109 Cologne, Germany; alexandra_schulz@hotmail.com (A.S.); fuchsp@kliniken-koeln.de (P.C.F.)

**Keywords:** hand burn, eschar removal, enzymatic debridement, bromelain, dermis preservation, scarring, functional and aesthetic outcome, comparison of enzymatic versus surgical debridement

## Abstract

*Background and Objectives:* Burn surgery on the hands is a difficult procedure due to the complex anatomy and fragility of the area. Enzymatic debridement has been shown to effectively remove burn eschar while minimizing damage to the surrounding tissue and has therefore become a standard procedure in many burn centers worldwide over the past decade. However, surprisingly, our recent literature review showed limited valid data on the long-term scarring after the enzymatic debridement of the hands. Therefore, we decided to present our study on this topic to fill this gap. *Materials and Methods:* This study analyzed partial-thickness to deep dermal burns on the hands that had undergone enzymatic debridement at least 12 months prior. Objective measures, like flexibility, trans-epidermal water loss, erythema, pigmentation, and microcirculation, were recorded and compared intraindividually to the uninjured skin in the same area of the other hand to assess the regenerative potential of the skin after EDNX. The subjective scar quality was evaluated using the patient and observer scar assessment scale (POSAS), the Vancouver Scar Scale (VSS), and the “Disabilities of the Arm, Shoulder, and Hand” (DASH) questionnaire and compared interindividually to a control group of 15 patients who had received traditional surgical debridement for hand burns of the same depth. *Results:* Between January 2014 and December 2015, 31 hand burns in 28 male and 3 female patients were treated with enzymatic debridement. After 12 months, the treated wounds showed no significant differences compared to the untreated skin in terms of flexibility, trans-epidermal water loss, pigmentation, and skin surface. However, the treated wounds still exhibited significantly increased blood circulation and erythema compared to the untreated areas. In comparison to the control group who received traditional surgical debridement, scarring was rated as significantly superior. *Conclusions:* In summary, it can be concluded that the objective skin quality following enzymatic debridement is comparable to that of healthy skin after 12 months and subjectively fares better than that after tangential excision. This confirms the superiority of enzymatic debridement in the treatment of deep dermal burns of the hand and solidifies its position as the gold standard.

## 1. Introduction

Our hands are key parts of who we are and how we interact with others. They are capable of a wide variety of functions, such as touching, feeling, holding, and working. Hands are constantly visible, which is why aesthetically deformed hands often carry a debilitating social stigma [1]. Guy Foucher’s statement, “hand surgery is also aesthetic surgery”, hence became a central aspect in burn treatment [2]. Many patients who have suffered a hand burn injury experience long-term scarring, pigmentation, surface irregularities, and functional deficits. Therefore, long-term hand function and aesthetics should be the main focus of hand burn treatment, and efforts should not be spared in order to optimize the long-term outcomes [3]. 

Early debridement and immediate grafting is known to be the gold standard in burn wound management [4]. Surgical tangential necrectomy (standard deviation) was established in the 1970s as the standard of care for burn wound treatment by Dr Janzekovic [4]. For decades, burn surgeons have had a choice of techniques such as dermabrasion, hydrosurgery (Versajet), or use of the Weck knife to perform surgical tangential excision. Nevertheless, all of these techniques are, to some extent, traumatic and non-selective, causing damage to viable tissue and requiring skin graft transplantation in the majority of cases [5,6,7,8]. However, in many burn centers worldwide, tangential necrectomy and skin grafting is still the standard of care for burn wound treatment.

In 2012, NexoBrid was introduced in the European market. This novel enzymatic debriding agent has been found to fulfill the desire for selective burn wound debridement and resulted in a significant reduction in the need for autografting. Previous studies have shown that after enzymatic debridement (EDNX), the wound healing time may be slightly delayed by a few days [6]. However, it has been shown that the scar quality is good in facial burns [9]. In a previous study, we evaluated the acute treatment and early scarring after three months in partial-thickness to deep dermal hand burns following enzymatic debridement. Although EDNX has become a standard procedure in many burn centers worldwide during the past ten years, surprisingly, our recent extensive search of the literature showed limited valid data on the long-term scarring after the enzymatic debridement of the hands. Shoham et al. conducted a systematic review in 2023, finding 103 relevant studies, of which 34 were found eligible [10]. Among these, there were several studies investigating the long-term outcomes of hand burns after enzymatic debridement. However, the scar quality was only assessed subjectively using questionnaires. Corrales-Benitez et al. found normal and improved results in DASH and MHOQ [11]. Pertea et al. showed superior scar quality regarding the VSS [12]. Rosenberg et al. conducted a multicenter RCT in 2014 and found superior long-term outcomes; however, only the MVSS was used [6]. In summary, reports on long-term scar quality following EDNX are rare in the literature, and objective scar evaluations of the hand do not exist in the literature. Therefore, we processed our data from 10 years ago to conduct a study with the aim of filling this knowledge gap.

## 2. Materials and Methods

NexoBrid is an orphan drug for the enzymatic debridement of burn wounds that is based on lyophilized, purified proteolytic proteins with bromelain, a pineapple plant-stem derivative [13]. Proteolytic enzymes are mixed with an inert carrier gel for easy handling [6]. Previous studies have shown that NexoBrid effectively dissolves burn eschar within four hours of application without harming non-burned, viable tissues [14]. One of the main advantages of NexoBrid in terms of post-debridement and wound healing is that it leaves enough non-injured dermis that can re-epithelialize spontaneously, thereby significantly reducing the need for autografting [5,6]. 

This study followed all of the guidelines for experimental investigation with human subjects required by the Ethical Review Committee at the University of Witten-Herdecke in Germany (protocol nr. 185/2014). This study was conducted at a leading German academic medical burn center, and complete informed consent was obtained from all patients. 

### 2.1. Patient Selection

**Figure 1:**
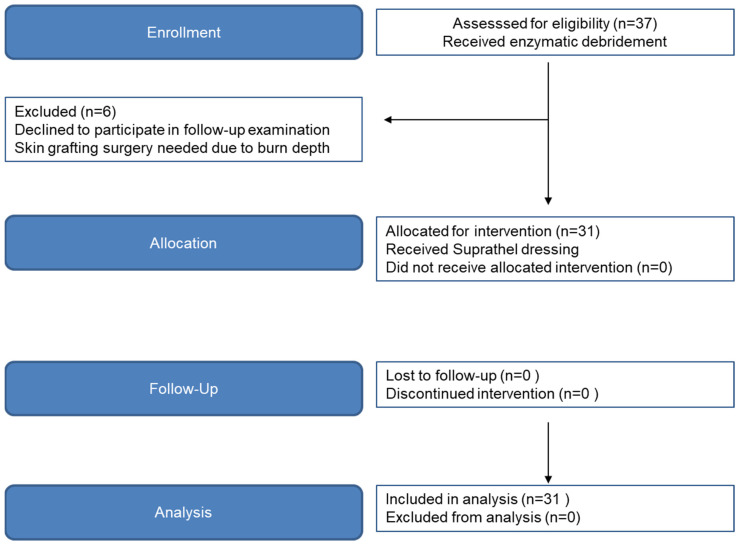
Flow diagram of CONSORT study phase progression.

Upon admission, hand burns were assessed by a single senior burn surgeon. Clinical characteristics, such as color, capillary refill, skin pliability, sensation, blistering, and vascular thrombosis, were checked before evaluation [15]. If partial-thickness to deep dermal hand burns were identified and the study criteria were met, patients were included in the study. In total, 46 patients were included. In terms of randomization, we placed the first 15 patients into the surgical excision and skin grafting group, and placed the following 31 patients in the EDNX group, when we adopted Nexobrid as the standard treatment (Figure 1). The contralateral uninjured hand of patients in the EDNX group additionally served as an intraindividual control group. Inclusion criteria were as follows: (a) adults aged 18 years or older of either gender, in good physical condition; (b) deep dermal thermal burns of the hand(s) caused by fire/flame or contact; (c) burn wound(s) requiring surgical eschar excision and/or escharotomy; (d) patient consent for NexoBrid treatment and mandatory follow-up examinations; and (e) wound area(s) of 0.5% or more of total burn surface area (TBSA). The following were grounds for exclusion: (a) lack of consent for required therapy and follow-up examinations; (b) pregnancy or nursing; (c) history of allergy and/or known sensitivity to pineapples, papaya, bromelain, or papain; (d) skin injuries caused by long-term corticosteroid therapy; (e) blood clotting dysfunction; and (f) pre-enrollment use of cerium nitrate/silver sulfadiazine or silver nitrate cream (g) wounds after debridement requiring skin grafting [15]. Patients’ characteristics, such as comorbidities, injury severity score (ISS), and skin type were also documented and did not differ significantly between the groups (Table 1).

### 2.2. Surgical Excision and Skin Grafting

The primary focus of this study was to systematically investigate the outcomes subsequent to enzymatic debridement. In order to elucidate the substantial variance in treatment efficacy quantitatively, we opted to incorporate the last 15 patients who underwent conventional surgical therapy, prior to the general transition to enzymatic debridement in our clinic as the standard of care in these cases, as the control cohort in this study. Thus, one-third of the study population underwent conventional surgical therapy, while two-thirds were subjected to enzymatic debridement. Between January 2013 and December 2013, 15 patients with partial-thickness to deep dermal hand burn wounds were treated with surgical excision followed by skin grafting. 

Following the initial wound cleansing step in our emergency department, fatty gauze was applied, and dressing was performed. In contrast to enzymatic debridement, a surgical procedure was scheduled for the next day. The operation was conducted under general anesthesia. The split-thickness skin graft was harvested from the lateral thigh and meshed at a ratio of 1:1.5 onto the previously debrided wound using Weck knives and secured with absorbable sutures. Patients were admitted for inpatient care. The first dressing change, involving the removal of the bolster dressing, occurred on the fifth postoperative day. In cases of significant split-thickness graft loss, a repeat transplantation was carried out in a similar manner. Patients were discharged for outpatient follow-up upon achieving stable wound conditions.

### 2.3. Enzymatic Debridement

**Figure 2:**
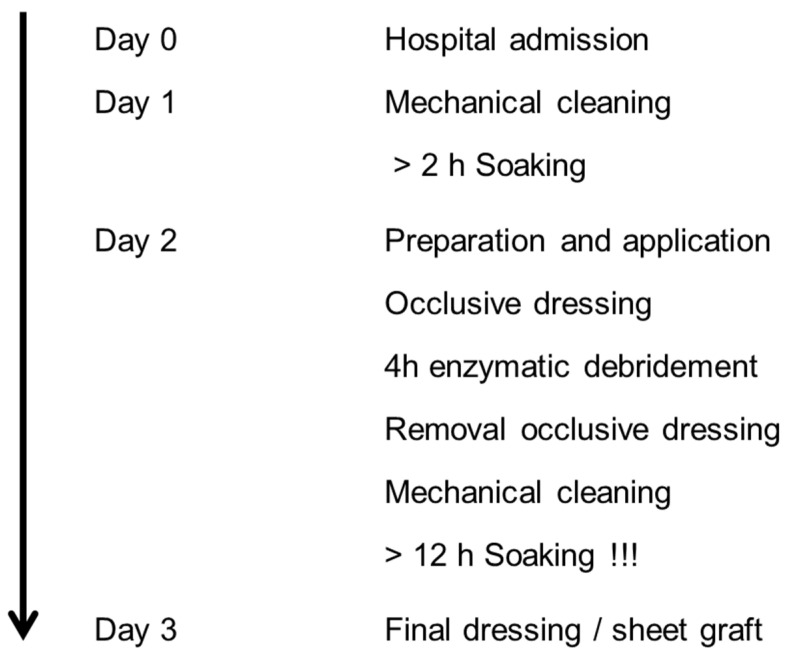
Standard of care: enzymatic debridement treatment.

**Figure 3:**
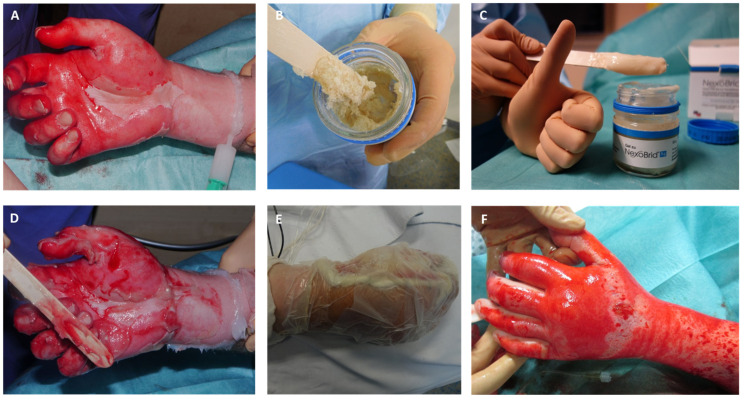
Process of enzymatic debridement of deep dermal hand burn, (**A**) Mechanical cleaning of wound bed after pre-soaking and creation of debridement border by applying paraffin; (**B**) homogenization of Nexobrid© substrate; (**C**) homogenized Nexobrid© substrate ready to use; (**D**) application of Nexobrid© to the wound bed; (**E**) occlusive dressing during process of enzymatic debridement; (**F**) wound bed after removal of the occlusive dressing showing selective debridement: punctate bleeding and white dermis.

Between January 2014 and December 2015, 31 patients with partial-thickness to deep dermal hand burns were enrolled in the current study. The enzymatic debridement procedure was performed in accordance with our standard of care (Figure 2 and Figure 3) under plexus block or local anesthesia, as already described elsewhere [8]. Wounds were covered with Suprathel. Patients were discharged from hospital as soon as their pain and wound conditions had improved to a point where outpatient clinic care was sufficient. During this outpatient phase, external dressing changes occurred on a weekly basis. Suprathel dressing was maintained until the healing process was complete, and once it naturally detached, the healed but still vulnerable skin received treatment with ointments containing panthenol for the first three months. To ensure consistent levels of skin care and range of motion, the uninjured hand, which served as the control, also underwent physiotherapy and received ointment treatment. Patients initiated daily physiotherapy on the third day after admission to promote a full restoration of manual dexterity. Furthermore, patients who underwent EDNX and those who underwent surgical excision with skin grafting wore compression garments for their hands over a period of six months. This practice began immediately after the completion of the wound healing process.

### 2.4. Wound Evaluation

The wound closure time (defined as less than 5% remaining defect) and adverse events were evaluated. Patients were discharged from the hospital when the pain and wound situation was manageable for outpatient clinic care. External dressing changes were performed on a weekly basis. Suprathel dressing was left in place until healing was completed.

### 2.5. Twelve-Month Follow-Up Examination

**Figure 4:**
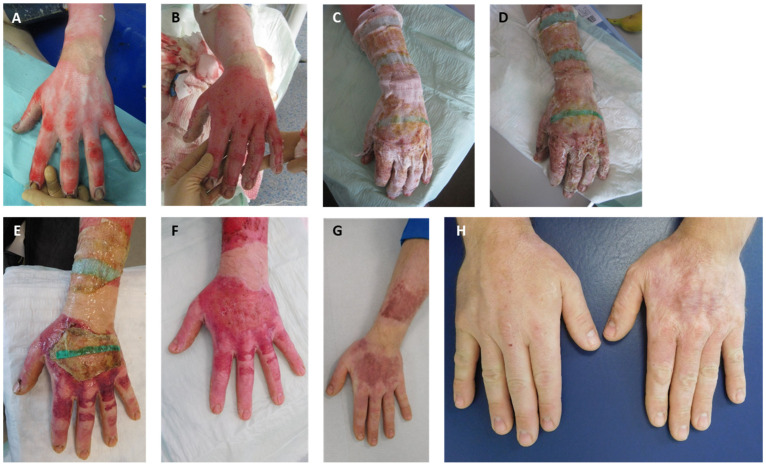
Injury pattern, acute treatment, and healing process: (**A**) burn on arrival; (**B**) wound bed after enzymatic debridement (punctate bleeding and white dermis); (**C**) wound after application of Suprathel©; (**D**) wound after 1 week; (**E**) wound after 2 weeks; (**F**) wound after 3 weeks; (**G**) outcome after 6 months; (**H**) aesthetic and functional outcome 12 months after injury with comparison to other side.

All subjects were asked to participate in a follow-up examination in our outpatient clinic after 12 months. Scars were documented through digital photographic imaging. Scar quality was evaluated subjectively using a questionnaire and objectively using devices (Figure 4).

### 2.6. Subjective Scar Evaluation 

Subjectively, scars were evaluated using the Patient and Observer Scar Assessment Scale (POSAS), the Vancouver Scar Scale (VSS), and the Disabilities of the Arm, Shoulder, and Hand (DASH) score. We compared the results of our study group (EDNX) with a second group of 15 patients who had been treated for partial-thickness to deep dermal hand burns using traditional surgical tangential necrectomy (standard deviation) methods. The hand burn wounds in the standard deviation group were assessed at admission by the same senior burn surgeon and were found to have similar burn depths and sizes.

The Vancouver Scar Scale (VSS), introduced by Sullivan et al. in 1990, was originally designed to evaluate burn scars [16]. It has been widely used in clinical studies to evaluate all types of scars, although it has been modified for this purpose. However, the VSS has been criticized for its low levels of validity and reliability [17,18]. The scale assesses “vascularity”, “pigmentation”, “thickness”, and “pliability” by assigning a score of 0 to 3 for each category [19]. A score of 0 indicates normal characteristics, while higher scores indicate poorer scar quality. The higher the score, the less the scar quality corresponds with normal characteristics.

The Patient and Observer Scar Assessment Scale (POSAS) was introduced by Draaijers et al. in 2004 [20] and is an improvement of previous scales because it considers both the patient’s and physician’s evaluations of the scar quality [21]. The observer assesses “vascularity”, “pigmentation”, “thickness”, “relief”, “pliability”, and “surface area” on a scale ranging from 1 (“like normal skin”) to 10 (“worst scar imaginable”) and by choosing one of three to five categories. Patients are asked six questions about pain, itching, color, stiffness, thickness, and irregularity, which are rated on a scale from 1 (“no” or “as normal skin”) to 10 (“yes”, “very different”). As a more recent tool, the POSAS has been accepted as feasible, effective, reliable, and valid in recent scar research [22,23]. 

The DASH score (Disabilities of the Arm, Shoulder, and Hand) is a tool used to measure the impact of upper limb impairments on a person’s daily life. It is calculated using a questionnaire that asks about problems with activities of daily living (such as dressing, bathing, and writing) and leisure activities (such as sports, hobbies, and playing musical instruments) because of upper limb impairments. The DASH questionnaire consists of 30 questions, each of which is scored on a 5-point scale, with higher scores indicating greater disability. The scores for each question are added to obtain a total score, which can range from 0 (no disability) to 100 (maximum disability) [24,25].

### 2.7. Objective Scar Evaluation 

For device-based scar evaluation, we focused on tools that have been scientifically validated and are reliable for assessing scar formation in burn wound follow-up. Objective scar evaluation was performed by determining melanin and erythema levels, skin elasticity, trans-epidermal water loss, and tissue perfusion. The measurement points were precisely defined using standardized digital photographic imaging and anatomical structures of the hand, and results were compared to untreated skin in the same patient in nearby areas or on the other hand. All examinations were performed in the same assessment room by a single experienced physician who was blinded to the treatment. Examinations took place in a standardized manner after the patient had been inactive for at least 20 min. To ensure stable results, all objective measurements were performed three times in succession.

#### 2.7.1. Mexameter^®^ MX 18

The Mexameter^®^ MX 18 (Courage + Khazaka electronic GmbH, Cologne, Germany) is a device that can measure the main components of skin color, erythema, and melanin in vivo and non-invasively based on the absorption and reflection of light emitted by the skin [26,27,28,29,30,31]. 

#### 2.7.2. Cutometer^®^ Dual MPA 580

The Cutometer^®^ dual MPA 580 (Courage + Khazaka electronic GmbH, Germany) precisely measures elasticity and other biomechanical parameters of the skin in vivo using a suction technique [32,33,34].

#### 2.7.3. Tewameter TM300

The Tewameter TM300 (Courage + Khazaka electronic GmbH, Germany) emits a beam of light at a specific wavelength, which is absorbed by the skin to a greater or lesser extent depending on its moisture content. The Tewameter then measures the amount of light that is absorbed and calculates the moisture content of the skin, which is often expressed as a percentage. Higher values indicate that the skin is more hydrated, and lower values indicate that it is drier. An intact physiological barrier function of the skin is essential for maintaining sufficient moisture in the outer cell layers of the skin. Therefore, trans-epidermal water loss is an important aspect of scar quality and skin recovery [30,35,36]. 

#### 2.7.4. O2C (Oxygen to See Device) 

The oxygen to see device (O2C; LEA Medizintechnik, Giessen, Germany) enables the evaluation of tissue oxygen saturation, hemoglobin level, and microcirculation through the continuous transmission of different wavelengths of light (839 nm and 500–800 nm). The light is dispersed on the skin’s surface and measured using the O2C device, which uses the data to calculate blood flow, hemoglobin oxygenation (SO_2_), and the relative amount of hemoglobin (rHB) [37,38,39].

### 2.8. Statistical Analysis 

We used Excel spreadsheets (Microsoft Office 2013; Microsoft Corp, Redmond, WA, USA) and SPSS v21 (SPSS Inc [IBM], Chicago, IL, USA) for data management, analysis, and chart design. We checked all data for completeness and accuracy before analysis and evaluated the data prospectively. We used the Wilcoxon test for pairwise comparisons and considered statistical significance to be *p*-values < 0.05. We used box plots to provide a comprehensive overview of the raw data.

## 3. Results

### 3.1. Enzymatic Debridement

In summary, 31 partial-thickness to deep dermal hand burn wounds (17 on the right hand and 14 on the left hand) were debrided enzymatically between January 2014 and December 2015. The mean TBSA was 13.18%, and the mean treated BSA as far as the hand was concerned was 1.12%. There were three female patients, and the rest were male, with ages ranging from 16 to 63 years old (mean age: 40 years; standard deviation: 11.70). All injuries were flame burns, and all wounds healed spontaneously after enzymatic debridement, with a mean healing time of 23 days (min 10 days, max 39 days).

### 3.2. Surgical Excision and Skin Grafting

Between January 2013 and December 2013, 15 patients with partial-thickness to deep dermal hand burn wounds were treated with surgical excision followed by skin grafting. The mean TBSA was 28.73%, and the mean treated BSA as far as the hand was concerned was 1.25%. All injuries were flame burns. The mean healing time was 32 days (min 11 days, max 80 days).

### 3.3. DASH Questionnaire Result 

In the current study, we found a mean DASH score of 7.15 (min, 0; max, 56,67; standard deviation, 16,02). Only 12.9% of the patients had a DASH score higher than 16, with scores ranging from 39.17 to 56.67, indicating severe disabilities. The best average answer was for question 4, “To prepare a meal”, with an average rating of 1.13. Only four patients reported minor difficulties preparing a meal. The worst rating was for question 23, “Did you feel limited in your work or daily life by problems with your hand, shoulder, or arm in the last week?” The average rating for this question was 1.48, with 77.4% of the patients answering “no, not at all.” A total of 9.7% reported minor limitations, 3.2% were moderately limited, 6.5% felt severely limited, and 3.2% felt that their limitations were severe. Other complaints were classified as low to minor, including shoulder, arm, and activity-related pain (mean rating: 1.48; min: 1; max: 5; standard deviation: 1.029) and tingling in the hand (mean: 1.55; min: 1; max: 4; standard deviation: 0.925). The patients also reported minor problems with weakness (mean: 1.32; min: 1; max: 5; standard deviation: 1.013) and stiffness (mean: 1.32; min: 1; max: 5; standard deviation: 1.013). Additionally, the patients reported relatively few problems with insomnia (mean: 1.32; min: 1; max: 3; standard deviation: 0.702. Furthermore, the patients generally did not report feeling less confident or useful (mean: 1.26; min: 1; max: 4; standard deviation 0.903).

### 3.4. POSAS (Patient/Observer) and Vancouver Scar Scale 

In our patient group, the mean POSAS score was 20.84 (min 7, max 49, standard deviation: 10.3) for the patient scale and 17.06 (range: min 6, max 31; standard deviation: 6.43) for the observer scale. In the comparison group of surgically treated patients, the mean POSAS score was 32.93 (min 11, max 60; standard deviation: 13.51) for the patient scale and 26.2 (min 9, max 36; standard deviation: 6.64) for the observer scale.

According to the POSAS scale, the patients rated their scars on a scale ranging from 1 (normal skin) to 10 (worst scar imaginable). On average, the scars from wounds treated with EDNX were rated as having minor pain (mean: 1.87), minor itching (mean: 2.71), and moderate in terms of color difference from normal skin (mean: 3.71). Stiffness (mean: 3.00), thickness (mean: 2.87), and irregularity (mean: 3.19) were all rated as minor to moderate. In comparison to the group treated with traditional surgical tangential debridement (SDT), the scar quality regarding stiffness, thickness, and irregularities was significantly superior (see Table 2).

According to the POSAS scale, the observer evaluated his patients’ scars on a scale from 1 (normal skin) to 10 (worst scar imaginable). He found that, in general, all categories for the EDNX-treated burn wounds of the hand ranged between minor and moderate. In comparison to the SDT-treated burn wounds of the hand, he found that the scar quality for the EDNX group was significantly superior for six out of seven categories (vascularity, thickness, relief, pliability, surface area, and overall opinion) (see Table 3).

According to the Vancouver Scar Scale, the majority of scars from EDNX-treated burn wounds of the hand tended to be pink (slightly increased in local blood supply) and supple (flexible with minimal resistance). The same findings were made in the comparison group after the traditional treatment. However, there was a significant difference found for pigmentation and scar tissue height for both groups. While the scars after the EDNX treatment were equally hyperpigmented (43.4%) or hypopigmented (46.7%), the scars after the traditional treatment were only hypopigmented (86.7%). Approximately 10% of the scars in both groups showed normal pigmentation. While the height of the scars was normal in the majority of cases (70%) in the EDNX group, the majority of scars in the comparison group had increased height (66.7% <2) (see Table 4).

### 3.5. Objective Scar Measurement

There were no significant differences in terms of flexibility of the tissue and trans-epidermal water loss between the untreated skin and scars following the EDNX treatment. However, the scars following the EDNX treatment were found to present significantly enhanced blood circulation (O2C_rHb; *p* = 0.041) and a notable erythema (erythema; *p* = 0.04) in comparison to the untreated skin (Table 5).

## 4. Discussion

Partial-thickness to deep dermal hand burns can be difficult to treat because the tissue layers in the hand are thinner and more delicate than those in other parts of the body. Damage to vulnerable structures in the hand, such as tendons, nerves, and vessels, may occur during the process of cutting away damaged tissue. The scarring of these underlying structures can result in significant deformity. Therefore, any second- or third-degree burn to the hand is considered an extreme burn injury and requires the attention of a burn center despite the burn’s small surface area. [40,41].

Enzymatic debridement is an effective technique for selectively dissolving burn eschar and preserving as much healthy tissue and native dermis as possible, as has been reported in previous studies [6,8,14,42,43]. Several studies have been conducted to evaluate the efficacy and safety of enzymatic debridement in the treatment of hand burns [5,44,45,46,47,48,49,50]. 

In this study, we aimed to evaluate the long-term outcomes of partial-thickness to deep dermal hand burns regarding aesthetics and functionality. A literature review conducted on PubMed using the terms “enzymatic debridement”, “scar”, and “hand” yielded only seven results (see Table 6). One of these studies was a literature review that focused on the treatment of acute thermal burns to the hand [51], and one study was a retrospective study [52]. Five studies were prospective and included 18-90 partial-thickness to deep dermal hand burn wounds. The follow-up period ranged from 3 months to 4 years. However, the scar quality was solely evaluated subjectively using the Vancouver Scar Scale (VSS), Patient and Observer Scar Assessment Scale (POSAS), Disabilities of the Arm, Shoulder and Hand (DASH), Patient-Related Wrist Evaluation Score (PRWE-G), and Michigan Hand Outcomes Questionnaire (MHOQ) [6,11,43,45,52,53]. The goniometer was only used as an objective measurement method in one study [11]. This analysis shows that there is a scientific need to measure scar quality after enzymatic debridement using both objective and subjective measurement methods. The current study aims to address this gap by evaluating long-term scarring after the enzymatic debridement of hand burns using both subjective and objective measurement methods.

In this study, none of the patients in the EDNX group underwent surgery. We believe that our extensive experience in enzymatic debridement allowed us to accurately identify wounds that require surgery after enzymatic debridement. Wounds that were expected to require grafting were not included in this study as we intended to focus on scars resulting from prolonged wound healing through spontaneous re-epithelialization. In line with the current literature, we found that wounds in this study closed without complications in an average of 23 days. Previous studies in the literature have reported prolonged wound closure ranging from 23 days to 27/28 days [8,45,46,47,48].

Early experiences with enzymatic debridement for partial-thickness to deep dermal hand burns showed that the scar quality may be at least equal to that of surgical debridement [5,6,43,45]. In a previous study, we found that the aesthetic and functional outcomes of partial-thickness to deep dermal facial burns was surprisingly superior in the enzymatic debridement (EDNX) group compared to the standard debridement (SDT) group based on the same measurement parameters used in our follow-up study on hand burns. The EDNX group had better outcomes in terms of pigmentation, thickness, relief, pliability, surface area, stiffness, thickness, and scar irregularity despite prolonged healing times [9].

After enzymatic debridement, Cordts et al. evaluated the Disabilities of the Arm, Shoulder, and Hand (DASH) score in 16 partial-thickness to full-thickness hand burns and found scores within the lowest quarter of the range (23/100, range: 0–45) [45]. Corrales-Benítez et al. found mean DASH and Michigan Hand Outcomes Questionnaire (MHOQ) scores of 0.18 and 99.71%, respectively, with a minimum follow-up of 391 days in 90 partial-thickness hand burns treated with enzymatic debridement [11]. Cherubino et al. retrospectively evaluated 18 hand burns after enzymatic debridement and found that the mean DASH score at 6 months was 21 and was reduced to 11 at the final follow-up visit [52]. The DASH score cutoff scores are described as “no problem” for any score less than 15. A DASH score between 16 and 40 expresses “problem, but working”. The DASH score cutoff scores are described as “no problem” for scores less than 15, “problem but working” for scores between 16 and 40, and “unable to work” for scores higher than 40 [54,55]. In the current study, we found a mean DASH score of 7.15 (min: 0; max: 56.67; standard deviation: 16.02). Only 12.9% of the patients had a DASH score indicating severe disabilities. These results, in line with the current literature, suggest that the functional outcomes of enzymatic debridement for partial-thickness to deep dermal hand burns are generally promising.

The main goal of the patient and observer scale is to assess the severity of patients’ scar conditions. Cherubino et al. found mean POSAS scores of 14.2 and 16 for the observer and patient scales, respectively, in their retrospective study of 18 partial-thickness to full-thickness hand burns 6 months after enzymatic debridement [52]. In our patient group, we found mean POSAS scores of 20.84 and 17.06 for the patient and observer scales, respectively, indicating slightly more issues with scar complications in our study. However, our study was prospective and included a larger number of patients (31 patients) compared to Cherubino et al.’s study (18 patients).

When comparing our EDNX group to a group of 15 hand burns treated with surgical debridement, we found that the scar quality in the EDNX group was significantly superior in terms of stiffness (*p* = 0.013), thickness (*p* = 0.002), and irregularity (*p* = 0.002) according to the POSAS patient scale, and in terms of vascularity (*p* = 0.003), thickness (*p* = 0.005), relief (*p* > 0.001), pliability (*p* < 0.001), and surface area (*p* = 0.007) according to the POSAS observer scale (see Table 3 and Table 4).

These findings are in line with the results from a 12-month follow-up study comparing partial-thickness facial burns debrided enzymatically with facial burns of similar depth treated with surgical debridement. This study found EDNX to be superior in terms of stiffness (*p* = 0.023), thickness (0.011), and irregularity (*p* = 0.011) according to the POSAS patient scale, and in terms of scar thickness (*p* = 0.16), scar relief (*p* = 0.10), scar pliability (*p* = 0.01), and surface area (*p* = 0.004) according to the POSAS observer scale [9].

Corrales-Benítez et al. found a mean VSS score of 2.87 in 90 partial-thickness burned hands 391 days after enzymatic debridement [11]. Cordts et al. rated the overall scar quality as 6/14 (4–8) points on the VSS scale and noted that full-thickness wounds that healed after ED treatment often exhibited intense scar vascularization, discoloration, and an increased susceptibility to further trauma [45]. In the current study, the mean VSS was rated 1.97 (min: 1, max: 3, standard deviation: 0.56), indicating slightly superior scar quality. In a previous study, we evaluated scar quality after 3 months in a similar group of partial-thickness to deep dermal hand burns [43].

At that time, 67% of the scars were hyperpigmented, and 33% were hypopigmented. In the current study (see Table 5), after 12 months, an equal number of scars were hyper- and hypopigmented, and in 10% of the scars, the pigmentation had even returned to the normal skin color. When comparing our EDNX group to a group treated with surgical debridement (STD), we found that the scars in the EDNX group were significantly more often of normal height or slightly elevated, while the majority of scars in the STD group were hypopigmented after 12 months.

When comparing the current study to the 3-month study, we found that the blood supply had reduced in the EDNX group; the majority of scars (20%) had normal blood supply and 63.3% had slightly increased blood supply, while in the 3-month study, the majority of scars (61%) had significantly increased blood supply.

We found similar results for pliability; while the majority of scars (44%) were flexible with minimal resistance after 3 months, and the remaining scars were either firm or only giving way to pressure, in our current study, after 12 months, a significant percentage (23.3%) of scars in the EDNX group were normal in pliability. The majority of scars (43.3%) were flexible with minimal resistance. In summary, according to the VSS, it appears that the scars in the EDNX group reduced in blood supply, pliability, pigmentation, and height over time.

To the best of our knowledge, this is the first study to compare 12-month scar quality of partial-thickness to deep dermal burn wounds in hands following enzymatic debridement using both subjective and objective methods. We found that, compared to uninjured skin on the same patient’s hand, blood supply (*p* = 0.008) and erythema (*p* = 0.003) were still significantly increased after 12 months in the EDNX group (see Table 6). However, the skin flexibility, resistance, and moisture content of the scar tissue were found to be equal to those of the uninjured skin after 12 months in the EDNX group (see Table 6).

This study demonstrates that enzymatic debridement is an effective treatment for partial-thickness to deep dermal burns of the hand, resulting in good long-term aesthetic and functional outcomes. The presence of viable, native dermis that was not excised or debrided likely contributes to the impressive quality of the resulting scars, as they heal via spontaneous epithelialization. In contrast, surgical tangential excision might not only remove eschar but also viable tissue to a certain extent, leading to a postoperative aggravation of burn depth. Previous research suggests that not only enzymatic debridement, but also tissue-preserving and gentle debridement strategies in general lead to improved aesthetic and functional outcomes for scars. Leaving a thin layer of native dermis to epithelialize is likely to result in a more satisfactory scar quality [56,57,58].

## 5. Conclusions

Based on the results of this study, it appears that enzymatic debridement may lead to better scarring outcomes in hand burns compared to traditional surgical debridement. However, the treated wounds showed increased blood circulation and erythema at 12 months post-treatment. Further research may be needed to fully understand the long-term effects of enzymatic debridement on scarring and other outcomes in patients with hand burn.

## 6. Study Limitations

The study has some noteworthy limitations. Firstly, it is worth mentioning that the extensive experience and prior knowledge of our department in handling Nexobrid may have, to some extent, contributed to the positive outcomes after enzymatic debridement. Given the significant increase in global burn centers’ experiences with enzymatic debridement over the past 10 years, it can now be assumed that all burn centers can achieve similarly favorable results, eliminating any potential bias. Furthermore, it was not possible to achieve blinding between the burn wound areas and untreated skin and between the enzymatic and surgical debrided hands. Additionally, the EDNX and standard deviation group hands showed slightly diverse BSA. To upgrade the information quality, theoretically, conducting randomized trials in an intraindividual setting would be the most effective way to confirm our findings. However, due to ethical considerations, we believe that conducting such studies is not advisable, since we are convinced that EDNX treatment is superior to standard deviation treatment regarding the preservation of functional tissue. We decided against such a study design due to ethical reasons. Furthermore, it is worth noting that other scientists who may not share our level of support for enzymatic debridement and lack the experience to use it regularly might not face the same ethical dilemmas. In any case, we believe that future studies should incorporate a larger number of patients with hand burns to further validate these findings.

## Figures and Tables

**Table 1:** Patients’ characteristics in the standard deviation group and the EDNX group.

Patient Number	Treatment	Age	Comorbidities	Injury Severity Score ISS	Third-Degree Burn (%)	TBSA (%)	Fitzpatrick Skin Phototype
Patient 1	standard deviation	28	nicotine abuse	4	0	7.5	II
Patient 2	standard deviation	28	nicotine abuse	4	0	7.5	II
Patient 3	standard deviation	29	pollinosis	25	38	78	II
Patient 4	standard deviation	41	none	25	25	59	II
Patient 5	standard deviation	25	none	9	0	24	III
Patient 6	standard deviation	25	none	9	0	24	III
Patient 7	standard deviation	55	rheumatoid arthritis, hypertension,	9	0	13	II
Patient 8	standard deviation	55	chronic lumbar pain syndrome	9	0	13	II
Patient 9	standard deviation	52	alcohol abuse	9	0	17	II
Patient 10	standard deviation	28	none	25	22	51.5	II
Patient 11	standard deviation	56	nicotine abuse	9	0	12	III
Patient 12	standard deviation	56	nicotine abuse	9	0	12	III
Patient 13	standard deviation	18	none	4	0	6	II
Patient 14	standard deviation	48	hypertension, cholezystolithiasis	16	10	32	II
Patient 15	standard deviation	48	shoulder-arm syndrome	16	8	32	II
Patient 1	EDNX	36	none	4	0	6.5	IV
Patient 2	EDNX	15	none	4	0	9	II
Patient 3	EDNX	43	suicidality, nicotine abuse	25	25	43.5	II
Patient 4	EDNX	43	nicotine abuse	25	12	43.5	II
Patient 5	EDNX	38	hypertension	4	0	6.5	II
Patient 6	EDNX	19	none	1	0	1	III
Patient 7	EDNX	51	ASS intolerance	1	0	1.5	II
Patient 8	EDNX	30	none	4	0	5	III
Patient 9	EDNX	30	none	4	0	5	III
Patient 10	EDNX	34	chronic gastritis	1	0	2	III
Patient 11	EDNX	45	hypertension	4	0	7.5	II
Patient 12	EDNX	62	none	1	0	2.2	II
Patient 13	EDNX	50	chronic gastritis	4	0	6.5	III
Patient 14	EDNX	48	none	1	0	0.6	II
Patient 15	EDNX	33	none	4	0	7	II
Patient 16	EDNX	29	nicotine abuse	1	0	1	II
Patient 17	EDNX	28	none	1	0	3	II
Patient 18	EDNX	47	none	1	0	4.5	II
Patient 19	EDNX	27	none	1	0	0.5	II
Patient 20	EDNX	22	nicotine abuse	1	0	4	II
Patient 21	EDNX	54	age-related hearing loss/presbyakusis	9	0	0.5	II
Patient 22	EDNX	43	none	16	12	24.25	II
Patient 23	EDNX	43	none	16	15	24.25	II
Patient 24	EDNX	52	alcohol abuse	9	0	17	II
Patient 25	EDNX	28	none	25	32	51.5	II
Patient 26	EDNX	44	nicotine abuse	1	0	1.5	II
Patient 27	EDNX	30	none	1	0	4	III
Patient 28	EDNX	30	none	25	28	65	II
Patient 29	EDNX	54	age-related hearing loss/presbyakusis	9	0	23.1	IV
Patient 30	EDNX	54	depression	1	0	23.1	IV
Patient 31	EDNX	33	nicotine abuse	4	0	9	II

**Table 2:** Evaluation based on POSAS patient scale 12 months after treatment: scars after EDNX versus untreated skin.

	EDNX Scar Evaluation				STD Scar Evaluation				
	Min	Max	Mean	STD	Min	Max	Mean	STD	*p* Value
Scar painful in the past weeks	1	7	1.87	1.61	1	9	3.33	2.47	0.006
Scar itching in the past weeks	1	8	2.71	1.95	1	9	4.80	2.68	0.009
Is the scar color different from the color of your normal skin at present?	1	9	3.71	1.72	2	6	4.20	1.57	0.313
Stiffness	1	7	3.00	2.00	1	10	4.73	2.34	0.013
Thickness difference from normal skin at present	1	8	2.87	1.82	2	9	5.07	2.25	0.002
More irregular than normal skin at present	1	8	3.19	1.96	1	8	5.40	2.13	0.002

**Table 3:** Evaluation based on POSAS observer scale 12 months after treatment: scars after EDNX versus untreated skin.

	EDNX Scar Evaluation				STD Scar Evaluation				
	Min	Max	Mean	STD	Min	Max	Mean	STD	*p* Value
Vascularity	1	5	3.00	1.10	2	5	4.07	0.96	0.003
Pigmentation	1	7	3.16	1.46	2	6	3.93	1.16	0.052
Thickness	1	6	2.87	1.52	1	7	4.40	1.68	0.005
Relief	1	6	2.81	1.19	2	12	5.40	2.23	0.000
Pliability	1	5	2.52	1.06	1	7	4.33	1.68	0.000
Surface Area	1	7	2.71	1.66	1	6	4.07	1.58	0.007
Overall Opinion	1	7	3.06	1.46	2	7	4.47	1.41	0.004

**Table 4:** Evaluation based on Vancouver Scar Scale 12 months after treatment: scars after EDNX versus untreated skin.

Vascularity (*p* = 0.575)		Pliability (*p* = 0.184)		Pigmentation (*p* = 0.007)		Height (*p* = 0.008)	
EDNX	STD	EDNX	STD	EDNX	STD	EDNX	STD
20% Normal	6.7% Normal	23.3% Normal	6.7% Normal	10% Normal	13.3 % Normal	70% Normal	26.7% Normal
63.3% Pink	80% Pink	43.3% Supple	40% Supple	46.7% Hypopigmentation	86.7% Hypopigmentation	26.7% <2	66.7% <2
16.7% Red	13.3% Red	23.3% Yielding	46.7% Yielding	43.4% Hyperpigmentation	0% Hyperpigmentation	3.3% >2 and <5	6.7% >2 and <5
		6.7% Firm	6.7% Firm				
		3.3% Banding	0% Banding				

**Table 5:** Objective scar evaluation 12 months after treatment: scars after EDNX versus untreated skin; measurement using O2C, Mexameter, Tewameter, Cutometer.

			Mean	STD	Min	Max	*p*
O2C	SO_2_	EDNX	60.9	24.1	0.8	95.0	0.188
		Healthy skin	65.1	21.2	14.0	93.0	
	rHb	EDNX	101.8	42.7	55.0	372.0	0.008
		Healthy skin	96.0	46.7	64.0	401.0	
	Flow	EDNX	71.0	73.6	5.0	303.0	0.915
		Healthy skin	61.1	47.1	6.0	238.0	
Mexameter	Melanin	EDNX	158.1	98.6	20.5	430.0	0.969
		Healthy skin	157.4	75.2	30.0	327.0	
	Erythema	EDNX	497.9	114.6	85.0	688.0	0.003
		Healthy skin	449.4	80.4	332.0	653.0	
Tewameter	Standard_AW	EDNX	0.2	0.1	0.0	0.5	0.878
		Healthy skin	0.2	0.1	0.0	0.5	
	SSWL	EDNX	3.0	2.0	0.3	8.3	0.284
		Healthy skin	2.7	1.9	0.4	8.1	
	Mean	EDNX	24.7	5.5	10.0	36.0	0.498
		Healthy skin	24.5	4.4	10.0	30.0	
Cutometer	R0	EDNX	0.9	0.4	0.3	2.0	0.100
		Healthy skin	1.0	0.4	0.1	2.1	
	R2	EDNX	0.8	0.2	0.0	1.0	0.203
		Healthy skin	0.8	0.2	0.0	1.0	
	F1	EDNX	0.2	0.2	0.0	1.0	0.939
		Healthy skin	0.2	0.1	0.0	0.6	

**Table 6:** Literature review on PubMed based on scar quality after enzymatic debridement of partial-thickness to deep dermal burn wounds on the hands.

Title	Author	Year	Study Aim	Wounds Included	Study Design	Burn Depth	Follow Up Period	Evaluation Tools
Evaluation of burned hand function after enzymatic debridement	Corrales-Benítez et al. [11]	2022	Evaluation of outcome	90 hands	prospective	partial thickness	3 months, 1 year	VSS, DASH, MHOQ, goniometer
Enzymatic Versus Traditional Surgical Debridement of Severely Burned Hands: A Comparison of Selectivity, Efficacy, Healing Time, and Three-Month Scar Quality	Schulz et al. [43]	2017	Comparison surgical versus enzymatic debridement	20 versus 20 hands	prospective	deep dermal - full thickness	3 months	VSS
Selective Enzymatic Debridement For The Management Of Acute Upper Limb Burns	Cherubino et al. [52]	2021	Describe the efficacy of treatment of upper limb burns with NexoBrid^®^ in a non-burn referral center	18 hands	retrospective			POSAS, DASH
Comparison of non-surgical methods for the treatment of deep partial thickness skin burns of the hand	Zacharecskij et al. [53]	2018	Comparison of 4 non-surgical methods for the treatment of deep partial thickness skin burns of the hand	87 hands	prospective	partial thickness	6 months	DASH, VSS
Acute Management of Thermal Hand Burns in Adults: A 10-Year Review of the Literature	Dargan et al. [51]	2021	Literature review acute thermal hand burns treatment		literature review			
A novel rapid and selective enzymatic debridement agent for burn wound management: A multi-center RCT	Rosenberg et al. [6]	2014	Does rapid enzymatic debridement with the debriding enzymeNexoBridTM (NXB) reduce need for surgery? Comparison surgical debridement	31 hands versus 41 hands	prospective	partial thickness-deep dermal	2-4 years	VSS
Enzymatic debridement for the treatment of severely burned upper extremities—early single center experiences	Cordts et al. [45]	2016	Proofing the results of enzymatic debridement in upper extremity burn wounds.	16 hands	prospective	partial thickness - full thickness	3 months	DASH, VSS

## Data Availability

The data are unavailable due to privacy restrictions.
